# Development of a Position Sensitive Neutron Detector with High Efficiency and Energy Resolution for Use at High-Flux Beam Sources

**DOI:** 10.6028/jres.110.069

**Published:** 2005-08-01

**Authors:** Diane M. Markoff, Vince Cianciolo, Chuck L. Britton, Ronald G. Cooper, Geoff L. Greene

**Affiliations:** North Carolina State University, Department of Physics, Raleigh, NC; Oak Ridge National Laboratory, Oak Ridge, TN; Oak Ridge National Laboratory, Oak Ridge, TN; University of Tennessee, Knoxville, TN

**Keywords:** cold neutrons, ionization chamber, MicroMegas detector, neutron detector

## Abstract

We are developing a high-efficiency neutron detector with 1 cm position resolution and coarse energy resolution for use at high-flux neutron source facilities currently proposed or under construction. The detector concept integrates a segmented ^3^He ionization chamber with the position sensitive, charged particle collection methods of a MicroMegas detector. Neutron absorption on the helium produces protons and tritons that ionize the fill gas. The charge is amplified in the field region around a wire mesh and subsequently detected in current mode by wire strips mounted on a substrate. One module consisting of a high-voltage plate, a field-shaping high-voltage plate, a grid and wire strips defines a detection region. For 100 % efficiency, detector modules are consecutively placed along the beam axis. Analysis over several regions with alternating wire strip orientation provides a two-dimensional beam profile. By using ^3^He, a 1/*v* absorption gas, each axial region captures neutrons of a different energy range, providing an energy-sensitive detection scheme especially useful at continuous beam sources.

## 1. Introduction and Discussion

The success of several proposed cold-neutron fundamental physics experiments depends on the ability to characterize the beam profile for understanding spatial and energy dependent systematics. Transmission experiments for both materials research and fundamental physics benefit from simultaneous characterization of the beam with downstream detectors. To achieve high count-rate profiles of the total beam area, a position and energy sensitive neutron detector must be capable of processing the high neutron source flux. Current good-resolution, position sensitive neutron detectors are limited in the flux they can process by intensity-dependent dead time. At present, there is no position sensitive neutron detector that can process neutron currents greater than 10^6^/s with a nearly 100 % efficiency over an active area of about 10 cm × 10 cm.

We are developing a neutron detector appropriate for high-flux sources in particular at the cold-neutron facility of the Spallation Neutron Source (SNS) [1] currently under construction, and at the High Flux Isotope Reactor (HFIR) [2] currently being expanded to include a very low-energy beam line, both located at the Oak Ridge National Laboratory (ORNL). We aim to design a detector that can collect the entire neutron beam on the order of 5 × 10^8^ cm^−2^ s^−1^ over a surface area of ~100 cm^2^ expected at these facilities.

Our detector design builds on the success of a segmented helium-3 ionization chamber and a neutron MicroMegas detector. The ionization chamber technique is employed to convert neutrons to charged particles in a large spatial region. The MicroMegas (MicroMesh Gaseous Structure) detector concept developed at the European laboratory, CERN [3], is used to convert charged particles to an electron shower that is subsequently detected on one-dimensional wire strip arrays or on two-dimensional pixels.

A segmented ionization chamber based on a helium-3 and argon gas mixture with coarse spatial and energy resolution was built for a preliminary fundamental physics measurement of the neutron spin rotation in a liquid helium target [4]. (For a description of this experiment, please see the article in the Weak Hadronic Interaction section of these proceedings.) Neutrons are detected through their capture on ^3^He producing a triton and proton: n + ^3^He → ^3^H + ^1^H with a *Q* value of 750 keV. These charged reaction products ionize the fill gas producing charged particles that are subsequently detected. In the segmented ionization chamber, a gas ratio of 0.5 bar ^3^He and 3 bar Ar produced 2 × 10^4^ ion pairs on average per neutron absorbed, that were collected on charge collecting plates divided into independent quadrants. The quadrants provided coarse position sensitivity with valuable count-rate information as a function of upper, lower, right and left sides of the beam. To improve the position resolution, we propose to use wire strips to collect the electron charge associated with an absorbed neutron.

Originally designed for charged particle detection and later modified for fast neutron detection, the MicroMegas detector concept has proven capable of processing a high charged particle rate with relatively fast recovery times to minimize dead time. Building on this success, a slow neutron MicroMegas detector has been developed by a team (including some of the authors of this paper) at ORNL [5],[6]. A solid neutron converter material of boron is placed on the aluminum drift electrode producing alpha particles that ionize the fill gas mixture of argon and methane. The electrons are accelerated to a nickel mesh grid in the conversion gap and multiplied in the amplification gap to produce an electron shower that is subsequently detected on pixels in a two-dimensional array.

This neutron imaging system will be the first position sensitive transmission monitor that can process relatively high neutron rates and provide timing information. The low energy neutron MicroMegas detector is optimized for performance at the SNS as an upstream beam monitor by absorbing 10 % or less of the neutrons while minimizing scattering effects that might disturb the beam [7]. The short drift path for the ions ensures low dead time for the expected count rates.

**Fig. 1:**
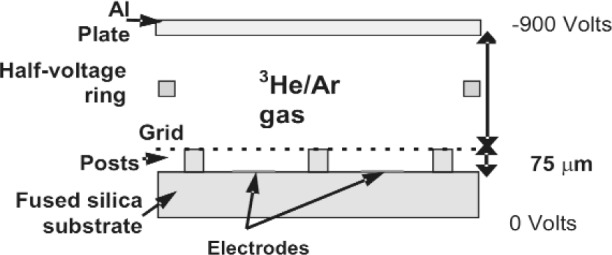
Schematic diagram of the proposed combined ^3^He ionization and MicroMegas neutron detector. The neutrons pass through the aluminum plate drift region electrode and are captured on the helium. The protons and tritons ionize the gas and the electrons are accelerated in the drift region toward the wire mesh and multiplied in the amplification region. The charge is collected on the electrodes that are one-dimensional arrays of wire strips oriented in one of two perpendicular directions. (Not drawn to scale.)

In Fig. 1, we provide a schematic drawing of our proposed detector that uses helium-3 to convert incoming neutrons to charged particles and detects the associated charge on wire strips. Our initial goal is for a 1 cm position resolution, using 5 mm wide strips located below a wire mesh that amplifies the electron charge in a localized region. The fill gas pressure of argon will be optimized for limiting the transverse range of the protons and tritons to maintain position sensitivity. The ^3^He pressure and drift length will be optimized for efficient conversion of neutrons and localization of charge in the drift region. For increased efficiency and processing of high count rates, we expect to collect the charge in current mode operation. Since fast timing is not critical for this initial application, the drift length can be larger than the few millimeters in a typical MicroMegas detector. A half-voltage ring provides electric field shaping over the drift distance.

**Fig. 2:**
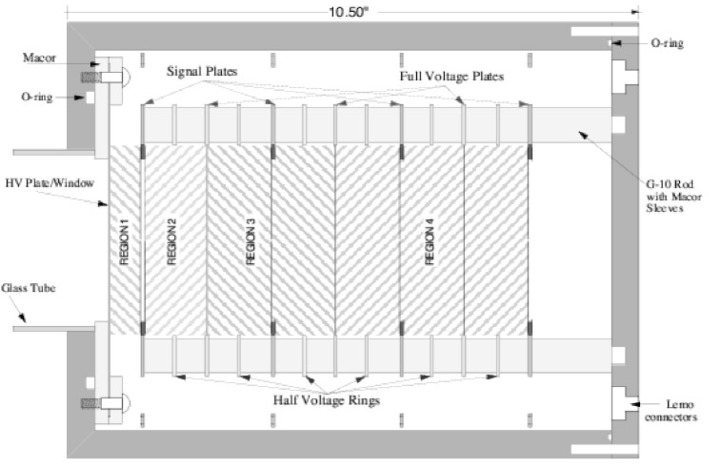
Cutaway view of the helium-3 segmented ionization chamber used in the preliminary measurement of the neutron spin rotation in helium. Note the four regions along the beam axis that are the four energy bins for detecting the neutrons. The fraction of the beam collected in each region was 33 %, 34 %, 24 %, and 8 % while the remaining 1 % of the beam was absorbed in a lithium material in the back of the detector. Taken from Ref. [4].

In the previous segmented ionization chamber, the collection plates and the high-voltage plates were repeated along the beam axis to divide the detector into regions. The plates were strategically located so that an appropriate electric field accelerated the charges to the correct collection plates. By using the ^3^He neutron absorber that follows the 1/*v* capture behavior, the distance the neutron travels in the detector before being captured increases with decreasing velocity. Each separate detection region collected neutrons of a different average velocity with the slowest ones detected in the first region and the faster ones detected in the following regions along the beam axis. A cross sectional diagram showing the four collection regions is given in Fig. 2.

Notice that the regions in the segmented ionization chamber increase in size further along the beam axis. This is necessary to maintain approximately equal neutron collection in each region with a single high-voltage and constant fill gas pressure. Note that the last two regions were summed to equal a third of the beam total as in the first two regions.

Similarly, we propose to construct a detector module from one set of high-voltage plates, wire mesh grid and array of wire strip electrodes as depicted in Fig. 1. Alternating modules will have alternating orientations of the wire strip arrays with the vertical orientation mounted on one side of the silica substrate and the horizontal orientation mounted on the other side, a module pair is naturally formed as two modules “back-to-back”. Analysis over multiple modules provides 2-dimensional spatial information of the neutron beam. Each detector module will be designed to absorb approximately 5 % of the neutron flux so that a series of 20 modules (or 10 module pairs) located along the beam axis absorbs the entire beam. As in the previous segmented ionization chamber, the high-voltage will be the same for each module and the fill gas pressures will be constant requiring varying drift distances to achieve equal neutron collection regions.

We are in the process of developing a 2 module prototype detector with 8 wire strips in each perpendicular orientation.

This position-sensitive neutron detector with energy separation will provide an easy to use and economical means of measuring the beam profile of an intense source to verify the neutron guide properties and study non-uniformities. This detector would serve as a beam intensity monitor located downstream of a transmission experiment, providing both spatial and energy information. A detector of this type would enable online, continuous monitoring of second order geometry-dependent beam characteristics that impose systematic limits on experiments that are sensitive to higher moments of the beam distribution [7]. (See for example the article on the measurements of neutron decay correlation coefficients in these proceedings.) For a continuous reactor source where time-of-flight techniques for energy dependent neutron detection are unavailable, this detector segmentation scheme enables neutron detection as a function of both geometry and relatively large, though useful, energy bins where the energy resolution strongly depends upon the segmentation structure.
